# SSVM: An Ultra-Low-Power Strain Sensing and Visualization Module for Long-Term Structural Health Monitoring

**DOI:** 10.3390/s21062211

**Published:** 2021-03-22

**Authors:** Suleman Khan, Jongbin Won, Junsik Shin, Junyoung Park, Jong-Woong Park, Seung-Eock Kim, Yun Jang, Dong Joo Kim

**Affiliations:** 1 Department of Civil and Environmental Engineering, Chung-Ang University, Seoul 06974, Korea; sulemank137@cau.ac.kr (S.K.); sac1721@cau.ac.kr (J.W.); jacoom1030@cau.ac.kr (J.S.); pjy5451@cau.ac.kr (J.P.); 2 Department of Civil and Environmental Engineering, Sejong University, 98 Gunja-dong, Gwangjin-gu, Seoul 143-747, Korea; sekim@sejong.ac.kr (S.-E.K.); djkim75@sejong.ac.kr (D.J.K.); 3 Computer Engineering and Convergence Engineering for Intelligent Drone, Sejong University, 98 Gunja-dong, Gwangjin-gu, Seoul 143-747, Korea; jangy@sejong.edu

**Keywords:** structural health monitoring (SHM), ultra-low-power, strain sensing and visualization module (SSVM), QR code, smartphone application, cloud database

## Abstract

Structural health monitoring (SHM) is crucial for quantitative behavioral analysis of structural members such as fatigue, buckling, and crack propagation identification. However, formerly developed approaches cannot be implemented effectively for long-term infrastructure monitoring, owing to power inefficiency and data management challenges. This study presents the development of a high-fidelity and ultra-low-power strain sensing and visualization module (SSVM), along with an effective data management technique. Deployment of 24-bit resolution analog to a digital converter and precise half-bridge circuit for strain sensing are two significant factors for efficient strain measurement and power management circuit incorporating a low-power microcontroller unit (MCU), and electronic-paper display (EPD) enabled long-term operation. A prototype for SSVM was developed that performs strain sensing and encodes the strain response in a QR code for visualization on the EPD. For efficient power management, SSVM only activated when the trigger-signal was generated and stayed in power-saving mode consuming 18 mA and 337.9 μA, respectively. The trigger-signal was designed to be generated either periodically by a timer or intentionally by a push-button. A smartphone application and cloud database were developed for efficient data acquisition and management. A lab-scale experiment was carried out to validate the proposed system with a reference strain sensing system. A cantilever beam was deflected by increasing load at its free end, and the resultant strain response of SSVM was compared with the reference. The proposed system was successfully validated to use for long-term static strain measurement.

## 1. Introduction

Civil structural failures in past years have increased the demand for structural health monitoring (SHM), which is the current key interest of the public to prevent future losses [1,2,3]. Aging civil structures mostly suffer catastrophic failures as the performance and condition degrade over time due to environmental factors, loads (dead/live), and design errors [4]. SHM provides the necessary quantitative details [5] that facilitate condition assessment for decision making, i.e., whether to demolish or maintain the structure. Proper countermeasures regarding maintenance increase serviceability along with the assurance of safety. Advanced sensor technology has enabled the use of precision devices for high-fidelity data acquisition. Displacement and strain are among the salient attributes for structural condition assessment [6,7,8,9,10]. However, factors such as power consumption, installation cost, and data management for the systems used in SHM are still under consideration [11,12,13]. For cost reduction, conventional wired sensors have been replaced with wireless sensors [14]. The wireless sensor networks (WSNs) consist of multiple nodes that have been deployed for civil infrastructure monitoring in the past [15,16,17]. However, these WSNs were confronted with certain limitations, such as power consumption and data management. For efficient power management, researchers have proposed hardware-based solutions [18,19] and solar energy harvesting [20] to increase battery life. However, these methods are still challenging due to inefficient power recharging in the absence of daylight. Furthermore, WSNs have a high probability of communication failure due to server or node failure [21], resulting in data mishandling.

Strain visualization is an emerging concept to address these issues for SHM and efficient long-term condition assessments of the structures. In the past, a wide range of mechanoluminescence (ML) materials have been used for strain visualization [22,23,24,25,26,27,28]. Among all ML materials, SrAl_2_O_4_:Eu (SAOE), SrAl_2_O_4_:Eu, and Dy (SAOED) were found to be most effective, because the light intensity of these materials is high, and can be seen in the daytime under mechanical load [29]. However, ML materials are not suitable for static monitoring because the emitted light intensity is dependent on the applied stress rate, not stress. In a recent study [30], the authors have developed a strain visualization system based on monochrome electro-luminance (EL) technology. EL panels are AC source-driven display units. The strain visualization using the EL panel was adequate compared to the use of ML material, but power-inefficient for continuous active display. EL panels also require a high-resolution camera, computation power, and complex algorithms to obtain strain responses from the light intensity. This increases the cost of an integrated strain sensing unit using an EL panel.

This study presents the development of an ultra-low-power strain sensing and visualization module along with a data management system that deploys display technology known as a monochrome electronic-paper display (EPD) [31]. EPD resembles the appearance of ink on real paper, because it reflects light instead of emitting it and has a wide view angle. The first EPD was developed in 1974 by Nicholas Sheridon, called Gyricon [32]. In 1998, Joseph Jacobson introduced another EPD that was called microcapsules [33]. After the critical review of Jason Heikenfeld [34] in 2011, regarding optical challenges for EPD, high total internal reflection was then involved for better display [35,36]. Currently available EPD panels have been used in many applications and have shown effective results [37]. The black-and-white EPD consists of micro-level electrophoretic capsules. Each capsule has positively charged white particles and negatively charged black particles suspended in a clear fluid. The capsules have electrodes connected on the top and bottom sides. The charge on the electrodes controls the movement of black and white particles to display the desired content. A high-fidelity and ultra-low-power strain sensing and visualization module (SSVM) acquires the strain response and encodes it in a QR code to display on the EPD. A QR code was used for automated data access and management instead of directly displaying strain response [38]. After displaying on the EPD, the QR code was then decoded, and data were uploaded to a cloud server. For this purpose, a smartphone application and cloud database were developed using open-source development platforms. For making module power efficient, a hardware design was also introduced. This study highlights the proposed design for SSVM, development of the SSVM prototype (hardware and software integration), and lab-scale experiment for validation and power consumption analysis.

The validation of the proposed system was performed with a custom-designed experiment. The setup consisted of a cantilever beam mounted on the table. Available laboratory resources (i.e., acrylic plates and batteries) were used as the load to deflect the beam. The strain was measured using SSVM and a reference strain measurement system with increasing load at the free end of the beam. The study included all the parameters related to strain sensing, power management, MCU selection, the process of visualization, and data storage.

## 2. Proposed Design for SSVM

**Figure 1:**
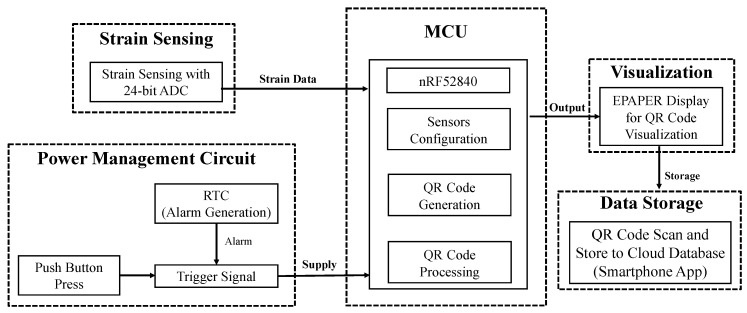
Block diagram of the strain sensing and visualization module (SSVM).

The strain sensing and visualization module (SSVM) was designed to monitor static strain responses and visualize them as a QR code on an electronic-paper display (EPD). Additional information such as the structure identity code (SID) and timestamp (TS) was also encoded in the QR code along with strain data. For data acquisition, the QR code image could be captured and decoded by the proposed smartphone application. The information can be uploaded to a cloud server-based data management system (see Figure 1).

SSVM uses a low-power microcontroller unit (MCU) with comparatively large SRAM. The function of MCU was the configuration of all serial peripheral interface (SPI) and I2C devices used in other blocks and data processing (voltage-to-strain conversion and QR code processing). For a power-efficient system, a power management circuit block was added. The function of this block is to control the power delivered to the MCU and other devices (e.g., ADC, EPD, etc.) based on the trigger-signal. The strain sensing block served for the acquisition of high-fidelity strain measurements with 24-bit analog-to-digital converter (ADC). The visualization block displays the data as a QR code on the EPD. For data storage, a smartphone application was developed to decode the QR code content and store them on a cloud database to monitor the structure.

### 2.1. Strain Sensing Circuit

A strain sensing circuit was designed for the acquisition of strain data under consideration of high-fidelity measurement standards. A half-bridge strain-to-voltage conversion circuit was deployed to avoid any drift in the strain response. Drift in strain response is a problem in conventional Wheatstone bridge circuits used for strain-to-voltage conversions. Strain gauges are sensitive to temperature change and initial strain heating phenomena; therefore, their resistance changes can cause a disturbance in Wheatstone bridge balance and result in a drift of strain response [39]. The use of another strain gauge instead of a resistor stabilizes the strain response. The bridge is precisely balanced because the effect of temperature is the same on both strain gauges.

**Figure 2:**
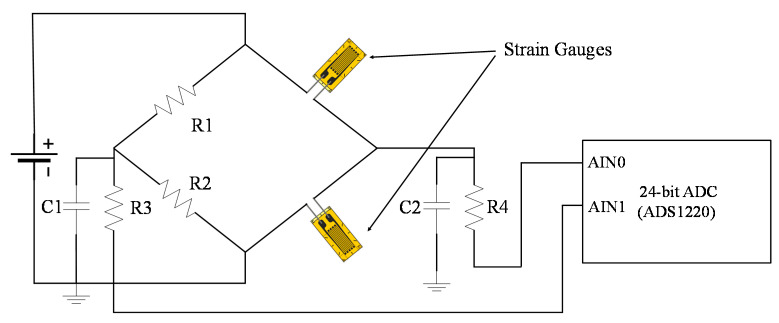
Half-bridge-based strain sensing circuit.

The differential voltage from the bridge circuit was then filtered and fed to 24-bit ADC to obtain strain response. Figure 2 shows the strain sensing circuit designed in SSVM for a single channel. R1 and R2 are 10 kΩ resistors to balance one-half of the bridge. The other half is balanced with two strain gauges (steel strain gauges, 350 Ω resistance). R3, R4, and C1, C2 were used for Resistor-Capacitor (RC) based low pass filtration of data. ADS1220, a 24-bit high-resolution ADC [40] from Texas Instruments was used to detect the differential voltage of the bridge, which can detect even small changes in the strain value. This voltage was then converted to the equivalent strain response inside the MCU.

ADS1220 is a low-power and low-cost ADC that provides a built-in programmable gain amplifier, noise filter (50–60 Hz), and voltage reference (2.048 V). There is no requirement of an external amplifier to detect small strain changes. It is ideally suited for resistive bridge circuits deployed for strain sensing. The Effective number of bits (ENOB) of ADC was 17.4 after performing several noise tests at 20 samples per second (SPS). The ADC with this ENOB provides 10 με resolution. The ADC has four single-ended and two differential channels. The ADC supports multiple modes for converting data; therefore, continuous conversion mode was selected, and data were acquired at 20 samples per second (SPS). The voltage conversion by ADC is given as follows [41]:

(1) $$Vout=\frac{ADC\_data*VFSR}{FSR}$$

where in *V_out_* is the differential voltage of the bridge circuit, *ADC_data* is the output values of ADC, *VFSR* is the ratio of the internal reference voltage (2.048 V) to programmable gain value, and *FSR* is the full-scale resolution of the ADC (2^24^). The voltage was then converted to the respective strain value using the relationship:

(2) ε=(Vout−offset)∗CF

In Equation (2), *ε* the is strain value, *offset* is the voltage value bias when the bridge is in a balanced state, and *CF* is the calibration factor for converting voltage to strain. A *CF* value of 576 was used for the conversion. An average value of strain was computed from 10 s of strain sensing and then encoded in the QR code.

### 2.2. Power Management Circuit

**Figure 3:**
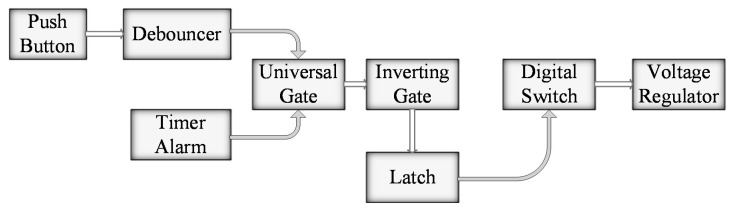
Functional block diagram of power management circuit.

For long-term monitoring of structures and to make the module power-efficient, a power management circuit was designed. The circuit controlled the power supplied by the battery to the MCU and other devices. A trigger-signal based power supply control was introduced, also known as power-saving mode, in SSVM. Figure 3 shows the functional block diagram of the power management circuit.

A push-button followed by a de-bouncer, MAX6816 [42], and a timer (RTC), DS3231 [43] from Maxim Integrated were used as sources of the trigger-signal. SSVM was activated from a push-button press or RTC alarm. A universal gate SN74AUP1G58 [44] from Texas Instruments (TI) was used to generate the combined response signal. RTC and push-button both were actively low; the universal gate inputs were adjusted to generate low output signal whenever one of them was activated. This low signal was then inverted to a high signal using a NAND gate, 74AUP1G00 [45] from Nexperia, configured as an inverting (NOT) gate. The high signal was then latched until the whole process of sensing and visualization was completed. SN74LVC1G373 [46] and TPS22860 [47] from TI are D-type latch and digital switch, respectively, deployed in this circuit. The digital switch was used to control the battery supply delivered to MCU and other devices. The source signals were latched, and a battery supply was delivered to initiate the process. After the QR code was displayed on the EPD, a reset signal from the MCU was generated to de-latch the trigger-signal and block the battery supply. Two 3.3 V voltage regulators, MCP1725 [48] from Microchip were also used to prevent voltage drops in the circuit. All the devices considered in the design of the power management circuit had low power consumption. A detailed power consumption analysis is stated in Section 5.

### 2.3. Microcontroller Unit (MCU)

Selection of the MCU was of great significance. The two primary considerations for MCU selection were low-power consumption and enough SRAM for processing and displaying QR code on EPD. These parameters were satisfied by adafruit nRF52840 bluefruit feather (Adafruit industries, NYC, USA), because it comes with large SRAM and low current consumption. The MCU processor has a 32-bit ARM^®^ Cortex™-M4 core with Arduino IDE support, running at 64 MHz clock speed, allowing the MCU to perform real-time processing in a fast and efficient manner. The MCU has 256 kB SRAM and 1 MB flash RAM for processing and displaying data as a QR code. The average current consumption of the MCU is less than 20 mA. nRF52840 supports both SPI and I2C communication protocols and has 7 digital and 6 analog input/output (I/O) pins. The analog I/O pins can also serve as digital I/O in this MCU.

### 2.4. Visualization of QR Code on EPD

**Figure 4:**
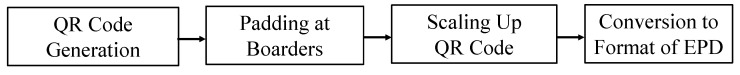
Processing and displaying QR code.

The EPD used was a 7.5 inch HD version in black-and-white display color by Waveshare electronics (Shenzhen, China) with a resolution of 880 × 528 pixels. The operating voltage of the EPD was 3.3 volts, and it used a four-wire SPI for communication with the MCU. The EPD has a large viewing angle (>170°). A QR code of version 3 with a resolution of 400 × 400 pixels was displayed on the EPD with a scan range of 1 m. Version 3 QR code can encode up to 55 bytes of data; strain response, SID, and TS (date and time) were easily encoded in this QR code version. The original size of the raw QR code generated inside the MCU was 29 × 29 modules. The modules are information content that make a black-and-white QR code. These modules were stored and upsized to 400 × 400 pixels inside the MCU. The final size of the QR code was 400 × 400 pixels, considering the SRAM usage of MCU by the whole process of upsizing the QR code and display it on EPD. Figure 4 shows the flow diagram of processing and displaying QR code on EPD.

The SRAM consumption for generating and displaying 400 × 400 QR code can be calculated as shown in Table 1.

The arrays are given in Table 2; raw_QR, padded_QR, and upsized_QR were two dimensional, and their data type was Boolean, while EPD_Format was a single-dimensional array of data type character. The total memory (SRAM) consumed by variable arrays to display QR code was 178 kB, which is 69.53% of the total SRAM of the MCU.

#### 2.4.1. QR Code Generation

QR code was generated using an open-source Arduino library [49]. Strain response, SID, and TS were converted to string format, and version (3) was specified to encode complete data in QR code. The output of the library was 29 × 29 modules. Zeros correspond to black pixels, and ones correspond to white pixels on EPD, so these modules were stored as pixels in array raw_QR. Due to the small size of the QR code generated (29 × 29), the further process proceeded to scale it up to 400 × 400.

#### 2.4.2. Padding at the Boarders

The approach used for scaling up the QR code was nearest-neighbor interpolation, owing to its low complexity and fast computation. When the raw QR code was directly scaled up, the output was disturbed from border regions due to quality reduction and high aliasing [50]. The raw QR code was padded with 4 pixels on each side to avoid this deformation and was saved in padded_QR (33 × 33). This array was then scaled up to 400 × 400 pixels.

#### 2.4.3. Scaling Up QR Code

The QR code generated was a binary image. Nearest-neighbor interpolation was the best and simplest approach to scale up, because no averaging of pixels is required in this technique. The algorithm to scale up a 33 × 33 image array to 400 × 400 using nearest-neighbor interpolation inside the MCU is stated as follows:

(3) $$\begin{array}{l} for(\mathrm{int}i=0;i<400;i++)\\ \{\\ \;for(\mathrm{int}j=0;j<400;j++)\\ \;\;\{\\ \;\;\;a=ceil(j*0.0825)\\ \;\;\;b=ceil(i*0.0825)\\ \;\;\;\mathrm{upsized}\_\mathrm{QR}=\mathrm{padded}\_\mathrm{QR}[b][a];\\ \;\;\}\\ \} \end{array}$$

In Equation (3), 0.0825 was the inverse of the scale factor, and the indexes of padded_QR were repeated in upsized_QR to upsize the 33 × 33 array to the 400 × 400 array of QR code. The upsized_QR was then converted to EPD format for display.

#### 2.4.4. Conversion to EPD Format

EPD accepts a single-dimensional array in hexadecimal format to display on the screen. The upsized_QR was converted to a single-dimensional hexadecimal array EPD_Format to be displayed on the EPD. Further using SPI communication protocol, the MCU transmitted these data to display on EPD.

### 2.5. Data Storage

**Figure 5:**
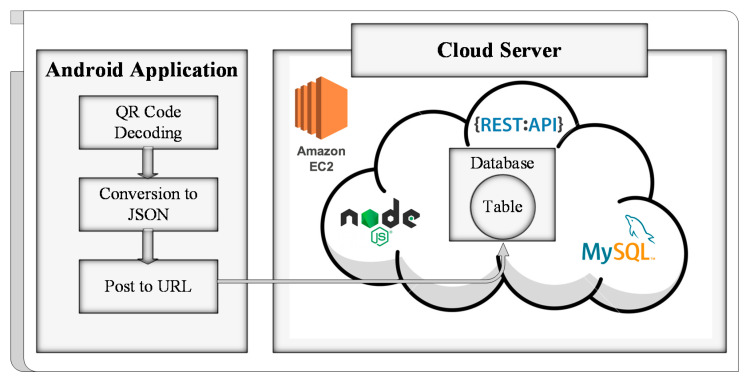
Basic framework of data storage.

The data were stored on a cloud server’s database (DB) when the QR code had been decoded. The process was accomplished with the development of a smartphone application and custom database for this task. The smartphone application was adopted for decoding QR code and uploading the content on the uniform resource locator (URL) of the table in the DB for the record. Representational state transfer (REST) application programming interface (API) was used to allocate a specific URL based on HTTP (hypertext transfer protocol). The POST request method was applied to upload data to a specified table in the DB of the server. Content of the QR code was converted into JSON format to upload it through the used method. The DB was created on a cloud server using the open-source database management service MySQL [51]. Amazon’s cloud computing platform elastic compute cloud (ec2) [52] service was employed as a server. Figure 5 shows the basic framework of data storage.

#### 2.5.1. Server Setup and DB Creation

An ubuntu instance (server) was first initialized on Amazon ec2. In this process, ports (access and listening) and protocols (HTTP, SSH, and TCP) were assigned for data transmission. The server was accessed through SSH (secure shell) client, MobaXterm [53], using the IP and key file obtained during the initialization process, then updated to install all the required packages for setting up a DB. MySQL’s (which was the first package installed) bind-address was modified to 0.0.0.0 to grant DB access to any localhost. After that, the port for access of MySQL was modified to access the port of the server. Upon installation of MySQL, access permission was granted to the localhost with a specific username and password. The localhost was then used to access MySQL of the server using the MySQL workbench [54] from specified credentials. In MySQL, a DB was created, and in DB, a table was generated to store the content of the QR code scanned by smartphone application after each measurement; the details are given in Section 4. The table consists of strain response, SID, and TS. A specific URL was assigned to DB after its creation. A few more packages (node js., body-parser, express, request) [55,56,57] were installed, and a Node.js script was run on the server to accept JSON data on the listening port via the post-request method.

#### 2.5.2. Smartphone Application Development

Android studio [58] was used to develop a smartphone application for uploading data to the server. The layout was designed, dependencies were included, and permissions were granted for required tasks. Permission included access to the camera and internet for application. An open-source QR code scanner library [59], developed by Yuri-Budiyev, was deployed for decoding QR code. The decoded content was then split and converted to JSON object format, because the DB was designed to accept JSON data. The data were then sent to the URL of the table in DB using post request generated by volley [60], an open-source HTTP library.

## 3. Development of SSVM Prototype

### 3.1. Hardware Integration

The prototype for SSVM assembled on Veroboard consisted of three parts. The first part included the triggering devices, power management devices, ADC, MCU, and connectors. The second part was designed for the strain-to-voltage conversion using a half-bridge circuit. Lastly, the visualization part consisted of the EPD and display driver circuit.

**Figure 6:**
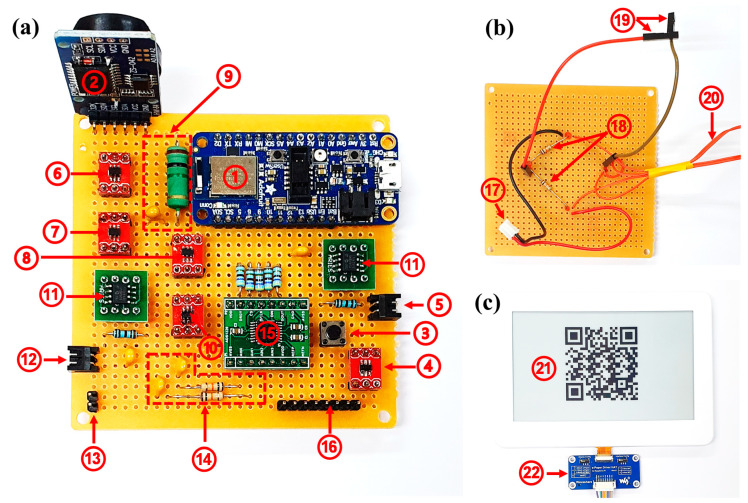
SSVM prototype: (**a**) triggering devices, power management devices, ADC, MCU, and connectors; (**b**) strain-to-voltage conversion; (**c**) visualization.

Figure 6 shows all parts that were used to make the prototype of SSVM. The components/devices of the prototype shown in Figure 6 are highlighted in Table 2.

The mechanisms of most of the components in Figure 6a have been discussed in Section 2. Additionally, the RC filter used to avoid noise in measurement had a cut-off frequency of 15.9 Hz (R3 = R4 = 10 kΩ, C3 = C4 = 1 μF) due to the data acquisition rate of 20 SPS configured on ADC. An RC delay element with a delay of 1 s (R = 1 MΩ, C = 1 μF) was added at the input pin (D) of the latch. The delay was required because the latch had two main inputs and a single output, which were latch enable (LE), input pin (D), and single-output (Q), respectively. Q was dependent on both LE and D. Therefore, if LE was high, Q followed D, and Q held the previous state of D while LE was low. In SSVM, we used the same signal (trigger-signal) at LE and D. When either RTC or the push-button were activated, it generated a low output from a universal gate that was further inverted to high by NAND configured as the NOT gate. The high input as available for a short time and should be latched. On the LE, this high signal was directly connected; while on D, it was delayed when the trigger-signal again became low. LE immediately entered into a low state, while D became low after LE due to delay. In this state, LE was low, and D resulted in a high Q. After completion of sensing and visualization, a high signal was generated from MCU for de-latching that turned off the supply for other components and MCU. Two voltage regulators were used; one for supplying voltage to the MCU and the other for strain sensing circuit and ADC. Figure 6b shows the strain-to-voltage conversion part of the strain sensing circuit. Figure 6c shows two devices; one is the EPD, and the second is the EPD driver circuit. The driver circuit was used for communication between EPD and MCU using the serial peripheral interface (SPI).

### 3.2. Software Integration

**Figure 7:**
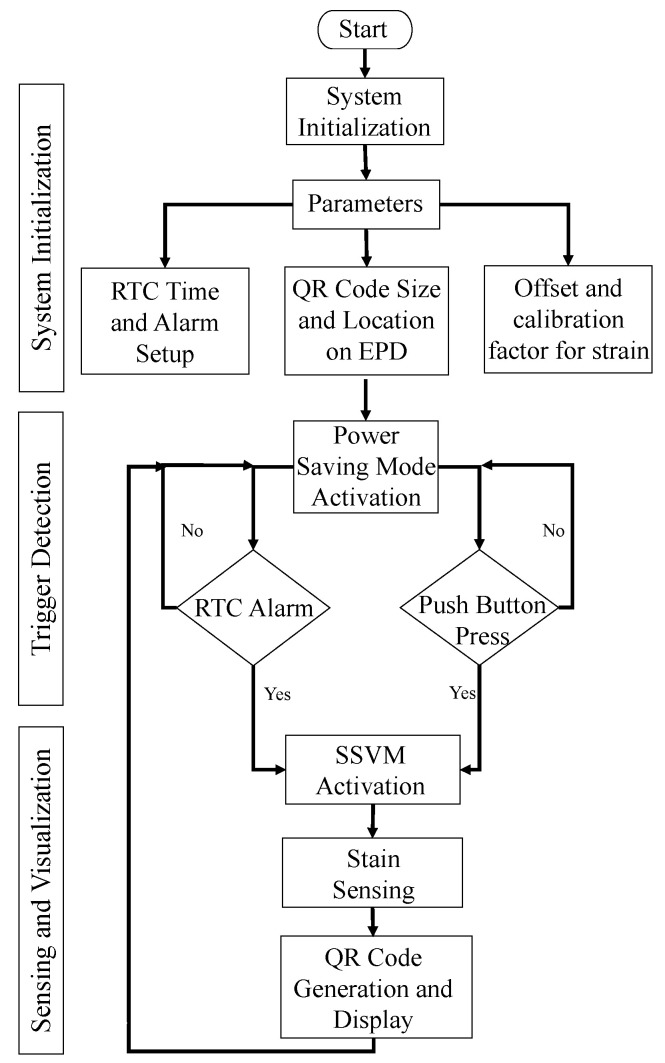
Integrated software framework flow for SSVM.

In addition to integrated hardware, a software framework was also developed to control SSVM through MCU. The MCU ha Arduino IDE support that made software integration easier due to open-source device driver programs [41,49]. The software integration involved three phases. The first was system initialization after the start; the second was trigger detection for activation; and the third was high-fidelity strain sensing and visualization. Figure 7 shows the complete flow of the integrated software framework for SSVM.

In the first phase (system initialization) parameters were set up, and device (RTC, EPD, and ADC) GPIOs were configured. The integrated program (Arduino script) that included parameter setup and device configuration information was uploaded to the MCU in this phase. RTC requires current time, date, and alarm trigger interval for TS generation and periodic sensing along with visualization. EPD requires the initial pixel coordinates (x = 240, y = 64 (centralized QR code on EPD)) and size of QR code (400 × 400) to be displayed. ADC requires the offset and calibration factor for converting voltage obtained from strain sensing circuit to equivalent strain response. In addition, ADC also requires data acquisition rate and channel configurations. After setting up all parameters of the devices through the MCU, the power saving mode of SSVM was activated, i.e., the second phase (trigger detection); the devices operated only when the trigger-signal was generated by RTC alarm or push-button press. Once SSVM was activated, it switched to the sensing and visualization phase, where its first task was to initialize strain sensing. The strain response was then embedded in a QR code that was further displayed on EPD. After completing the sensing and visualization phase, the MCU reactivates the power-saving mode of SSVM by sending a high signal from GPIO 13 to LE of the latch. There is no power on D that results in 0 voltage output at Q, and the digital switch is turned off. SSVM then waits for the next trigger-signal that again causes activation of the system. The process is continuous and highly effective for power-saving to develop a long-term monitoring system.

## 4. Evaluation of SSVM from Lab Scale Experiment

**Figure 8:**
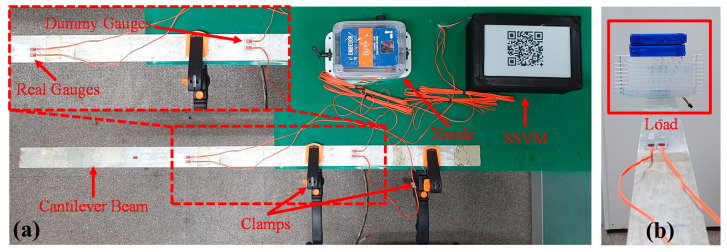
(**a**) Experimental setup for validation of SSVM; (**b**) loading.

A lab-scale experiment was designed for the validation of SSVM. The experimental setup consisted of a cantilever beam, table, clamps, loading materials/weights (acrylic plates and batteries), and steel strain gauges. The length and width of the beam were 1000 and 100 mm, respectively, and it was mounted on the table with the help of clamps such that one end was fixed on the table and the other was free to move. The free end was loaded with increasing weight to deflect the beam and measure strain response. Available lab resources were used to load the beam, including two materials, i.e., acrylic plates and batteries. As for strain measurement, a half-bridge circuit was used that involved the deployment of two strain gauges (dummy gauge and real gauge). Two pairs of gauges were attached to the beam, one for SSVM and the other for Xnode [61] that was used as a reference. Xnode, developed in 2017, offers high-fidelity strain measurements owing to its 24-bit ADC. The dummy gauges were attached to the fixed end of the beam, and real gauges were attached where deflection seemed to be maximum. Figure 8 shows the complete lab-scale experimental setup for validation of SSVM.

The data acquisition rate of SSVM was 20 SPS, and for Xnode it was 100 SPS. The sampling time for both the sensing boards was 10 s. The strain was sensed each time after the load was increased and the beam reached a static state. For comparison, the programmable gain for ADCs of both sensing systems was set as 1, and a calibration factor of 576 was used for steel strain gauges. The voltage offset was also considered in measurements for accurate comparisons. An average of 10 s measurements were computed to compare both SSVM and Xnode. The strain response from Xnode was stored in a memory card, while SSVM displayed it on the EPD as a QR code.

**Figure 9:**
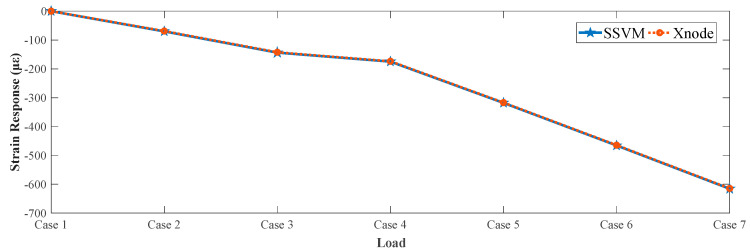
Strain response recorded by the proposed SSVM.

Table 3 shows the comparison of strain values obtained from both sensing systems at increasing load for validation of SSVM. At no load, the strain response was calibrated as zero. The load was then gradually increased in steps (case 2–7) to increase the deflection of the beam that resulted in increasing strain values. Root mean square error (RMSE) of strain response measured by Xnode and SSVM was computed as 1.15 με. The strain response plot of the comparison is also shown in Figure 9.

**Figure 10:**
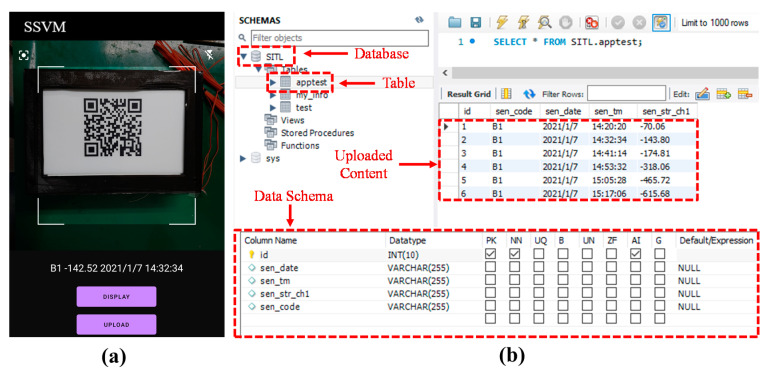
(**a**) Smartphone application; (**b**) cloud database (DB).

Along with strain sensing, data acquisition and management were performed through a smartphone application after each loading condition. The first step in data management is to scan QR code for the extraction of contents that included strain response, SID, and TS. Then, the next step is to upload the contents on a cloud DB. Figure 10a shows the user interface (UI) of the smartphone application.

The smartphone application developed was named “SSVM”. The UI of the application contains a scanning window, text viewer, and buttons. There are two buttons on the top screen; the one on the left side is for camera focus adjustment, and the other on the right side is for mobile flashlight operation. There is a text viewer under the scanning window that displays the content of the QR code when the display button is pressed. Under the display button, there is an upload button where its function is to upload the data decoded from QR code to the cloud DB. Figure 10b shows the MySQL workbench interface where the cloud sever was accessed using localhost. A DB named “SITL” was created in the cloud sever. Inside the DB, a table “apptest” was generated, and variables along with datatypes were defined through data schema. The QR code contents were uploaded to the URL of this table by POST method using the smartphone application. In the uploaded content, “id” represents a unique identifier for every uploaded content, “sen_code” is SID, “sen_date” and “sen_tm” are TS, and “sen_str_ch” is strain response.

## 5. Power Consumption Analysis of SSVM

SSVM performs sensing and visualization based on the trigger-signal. In this state, the current consumption is 18 mA, and the time duration is 35 s. In idle state (power saving mode), the current consumption of SSVM can be calculated from working devices (see Table 4).

Deploying six 3500 mAh (AA cell) batteries in parallel increased the overall capacity to 21,000 mAh. The battery life can be computed as [62]:

(4) $$Battery_{Life}=\frac{Capacity(mAh)}{I_{AVG}}\times 0.8$$

where 0.8 is a compensation factor for external and environmental conditions that affects battery life [63]. *I_AVG_* is the average current consumption and can be expressed using Equation (5) [62].

(5) $$I_{AVG}=I_{IDLE}(1-T_{s})+I_{SENSING}(T_{s})$$

Here, *T_s_* is sensing time (35 s), and *I_SENSING_* is sensing current (18 mA). Considering an operation once per month, SSVM has an *I_AVG_* equal to 338.137 μA and *Battery_Life_* of 49,684 h (5.75 years), making it ultra-low-power for strain measurements.

## 6. Discussion

SSVM is a state-of-the-art strain sensing and visualization tool that can be deployed for long-term condition assessments of civil infrastructure. Data can easily be managed using a smartphone application and cloud DB. The prototype proposed in this study is for single-channel strain response measurement and visualization. Future work would be upgrading the system to multiple channels and enabling structure deformation pattern prediction. Some constraints faced during development are the size of the QR code displayed and sampling time for strain measurement. The selected MCU, nRF53840, provides a large SRAM (256 kB) with less current consumption (20 mA), although the SRAM was not sufficient for this system. The maximum size of QR code that could be displayed using this MCU was 400 × 400, and at this resolution, 69.53% of SRAM is consumed. The integrated software program had an SRAM consumption of 85% that limited the sampling time for strain measurement. The current configuration allowed displaying a 3.5 inch QR code that could be scanned from a 1 m distance; sampling time did not have an impact while measuring static strain response. Considering these factors, the prototype was developed employing the same MCU; otherwise, MCU needs to be changed for displaying QR code with larger resolution.

Multiple nodes of SSVM or multi-channel SSVM can be deployed for the long-term monitoring of civil infrastructure such as bridges. Multi-channel strain can estimate the deflection and displacement of the structures [64,65]. This information can be utilized for efficient condition assessment of the structures by tracking changes in the overall shape of a structure and neutral axis.

## 7. Conclusions

This study presents the development and validation of a prototype for strain sensing and visualization along with an effective data management system. The sensing system enables long-term SHM with high-fidelity strain response measurements under low power consumption. An EPD that displays visualized information without power consumption was employed to visualize strain response as a QR code and storage before the next update. SSVM used a precise half-bridge circuit and 24-bit ADC that allowed efficient strain measurement. The system had low power consumption owing to a power management circuit, and low-power MCU and EPD. The power management circuit allowed activation of power-saving mode, where SSVM activated only while receiving a trigger-signal. Two sources were used for triggering, RTC and push-button. When the push-button was pressed or the RTC alarm was stimulated, SSVM initiated the strain sensing. After completion of strain sensing, SSVM embedded the strain response along with SID and TS in a QR code, processed the QR code, and displayed it on the EPD with a resolution of 400 × 400. Once the sensing and visualization process was completed, the power saving mode of SSVM activated again until the generation of the next trigger-signal. The MCU consumed a maximum of 18 mA current during the sensing and visualization phase. SSVM was validated by comparison of strain measurements with Xnode as a reference from a lab-scale experiment. A cantilever beam (fixed with table) was deflected by increasing load in steps at its free end. Strain response was measured from both sensing systems after each loading condition. An average of 10 s data were computed to compare static strain visualized by SSVM with the reference. The results showed good agreement between strain response acquired from both sensing systems, validating SSVM for SHM. The approach used for data management included the development of a smartphone application and a cloud DB. The QR code displayed on the EPD was scanned through the application, and decoded contents were uploaded onto MySQL cloud DB. Future work will seek the application of SSVM to long-term bridge girders and cable tension monitoring, incorporating cloud-based visualization.

## Figures and Tables

**Table 1:** Memory calculation.

Variable Array	Size (Dimension)	Size (Bytes)
raw_QR	29 × 29	841
padded_QR	33 × 33	1089
upsized_QR	400 × 400	160,000
EPD_Format	20,000	20,000
**Total**	178 kB	

**Table 2:** Components/device details.

Label	Components/Devices
1	nRF52840 (MCU)
2	ZS-042 (DS3231 RTC module)
3	Push-button
4	MAX6816 (Debounser)
5	Battery connector
6	SN74AUP1G58 (Universal gate)
7	74AUP1G00 (NAND gate)
8	SN74LVC1G373 (D-type latch)
9	RC delay element
10	TPS22860 (Digital switch)
11	MCP1725 (Voltage regulators)
12	Power connector for the strain-to-voltage conversion circuit
13	Differential voltage input connector
14	RC low pass filter
15	ADS1220 (24-bit ADC)
16	8-pin EPD driver connector
17	Connector for supplying power supply to the circuit
18	Bridge balancing resistors
19	Probes for output differential voltage
20	Strain leads for half-bridge configuration
21	EPD
22	EPD driver circuit

**Table 3:** Comparison between Xnode and SSVM.

Loading Materials		Xnode (με)	SSVM (με)
Case 1	No Load	0	0
Case 2	4 plates	−68.93	−70.06
Case 3	8 plates	−142.52	−143.80
Case 4	10 plates	−173.77	−174.81
Case 5	10 plates, 2 batteries	−316.94	−318.06
Case 6	10 plates, 4 batteries	−464.53	−465.72
Case 7	10 plates, 6 batteries	−614.5	−615.68

**Table 4:** Current consumption of SSVM during power-saving mode.

Device	Current Consumption (*I_IDLE_*)
ZS-042 (DS3231 RTC module)	200 μA
MAX6816 (Debounser)	6 μA
SN74AUP1G58 (Universal gate)	0.9 μA
SN74LVC1G373 (D-type latch)	10 μA
74AUP1G00 (NAND gate)	0.9 μA
TPS22860 (Digital switch)	0.1 μA
MCP1725 (Voltage regulators)	120 μA
**Total**	337.9 μA

## Abbreviations

SSVM	strain sensing and visualization module
EPD	electronic-paper display
MCU	microcontroller unit
EL	electro-luminance
SID	structure identity code
TS	Timestamp
Timer (RTC)	real time clock
ADC	analog to digital converter
ENOB	effective number of bits
SPS	sample per seconds
DB	database
URL	Uniform Resource Locator
